# Water associated zero maze: a novel rat test for long term traumatic re-experiencing

**DOI:** 10.3389/fnbeh.2014.00001

**Published:** 2014-01-17

**Authors:** Gilad Ritov, Gal Richter-Levin

**Affiliations:** ^1^Department of Psychology, The Institute for the Study of Affective Neuroscience, University of HaifaHaifa, Israel; ^2^Sagol Department of Neurobiology, University of HaifaHaifa, Israel; ^3^Psychology Department, University of HaifaHaifa, Israel

**Keywords:** WAZM, traumatic re-experiencing, amygdala, PTSD, rat model

## Abstract

Often, freezing and startle behaviors in the context of a previously experienced stress are taken as an indication of post-traumatic stress disorder (PTSD)-like symptoms in rats. However, PTSD is characterized by large individual variations of symptoms. In order to take into consideration the complex and long term distinctive variations in effects of trauma exposure additional behavioral measures are required. The current study used a novel behavioral test, the water associated zero maze (WAZM). This test was planned to enable a formation of an association between the context of the maze and an underwater trauma (UWT) or swim stress in order to examine the impact of exposure to the context which immediately precedes a stressful or a traumatic experience on rat's complex behavior. Rats were exposed to the WAZM and immediately after to an UWT or short swim. One month later rats were re-exposed to the context of the WAZM while their behavior was video recorded. Furthermore, c-Fos expression in the amygdala was measured 90 min after this exposure. The results of the current study indicate that the WAZM can be used to discern behavioral changes measured a long time after the actual traumatic or stressful events. Furthermore, the behavioral changes detected were accompanied by changes of c-Fos expression in the amygdala of exposed rats. We suggest that the WAZM can be used to model traumatic memories re-experiencing in rodent models of human stress-related pathologies such as PTSD.

## Introduction

Post-traumatic stress disorder (PTSD) is often conceptualized in terms of conditioned fear response with enhanced emotional memory mediated by a hyper-responsive amygdala (Gilboa et al., 2004; Francati et al., 2007). This conceptualization bears strong resemblance to the behavioral and neuronal system manifestations observed in fear-conditioned rodents (Elzinga and Bremner, 2002; Rau et al., 2005). Although there is no single widely accepted animal model of PTSD, fear conditioning in rodents can be used to model and elucidate different aspects of re-experiencing, a core symptom in PTSD, including the processing of fearful stimuli and the retrieval of emotional memory (Miller and McEwen, 2006). In laboratory rats, contextual fear conditioning can be easily induced by pairing neutral stimuli, such as context (conditioned stimulus-CS), with a fear-inducing stimulus, such as foot shock (unconditioned stimulus-US). Following this pairing, presentation of the context in which the conditioning was accomplished evokes a stereotypic behavioral fear response in the animal (Maren et al., 2013). The most common quantitative measurements of this behavior are calculations of freezing behavior and startle amplitude (Flint, 2003; Luyten et al., 2011; Maren et al., 2013). The manifestations of these behaviors have been shown to be accompanied by alterations in amygdala activation. Expression measurements of the immediate early gene c-Fos show that both the basolateral and central parts of the amygdala (BLA and CeA respectively) are activated during the retrieval of contextual fear conditioning (see Knapska et al., 2007 for review).

Since humans and animals display a range of responses to reminder cues of a traumatic event, solely measuring freezing and startle behavior might be insufficient. In order to take individual differences into consideration, multiple behavioral measures should be used (Cohen et al., 2004; Horovitz et al., 2012).

The “underwater trauma” (UWT) is a paradigm designed to model sudden and brief traumatizing experiences in rats (Richter-Levin, 1998). It has been shown to cause short and long term changes in anxiety-like behaviors as well as in various electrophysiological (Wang et al., 2000; Ardi et al., 2013) and biochemical (Sood et al., 2013) mechanisms. It was recently suggested that exposing animals to contextual reminders of the UWT may serve as an effective platform for elucidating neural mechanisms associated with traumatic re-experiencing (Ardi et al., 2013).

The current study used a novel behavioral test, the water associated zero maze (WAZM). This test enables the formation of an association between the context of the maze and water exposure. This association can be used to examine the impact of exposure to the context which immediately precedes a stressful or traumatic experience on rat's complex behavior and amygdala activation a long time after the exposure itself.

## Materials and methods

### Subjects

Twenty four male Sprague Dawley (SD) rats weighing 250–275 g (Harlan, Jerusalem, Israel) at arrival were habituated in the laboratory vivarium for 5 days. Animals were housed 2 per cage in a temperature-controlled (23 ± −1° C) animal quarters on a 12:12-h light-dark cycle (lights on 0700–1900 h). They had *ad libitum* access to standard rodent chow pellets and water.

Following the 5 days of acclimation to the laboratory vivarium all rats were randomly assigned to one of the following experimental conditions:

UWT (“UWT”; *n* = 8)—rats were exposed to 4 consecutive days of testing and short 30 s swim in the WAZM. On the 5th day, rats were exposed to testing and UWT stress in the WAZM. On the 30th day, rats were exposed to a contextual reminder of WAZM testing.

Swim (“Swim”; *n* = 8)—rats were exposed to 4 consecutive days of testing and short 30 s swim in the WAZM. On the 5th day, rats were exposed to testing and a longer 45 s swim in the WAZM. On the 30th day, rats were exposed to a contextual reminder of WAZM testing.

Control (“Control”; *n* = 8)—rats were exposed to 5 consecutive days of testing without water exposure in the WAZM. On the 30th day, rats were exposed to a contextual reminder of WAZM testing.

The study was approved by the ethics committee of Haifa University. Experiments were carried out in accordance with the Guidelines laid down by the NIH in the US regarding the care and use of animals for experimental procedures.

### The water associated zero maze

**Figure 1:**
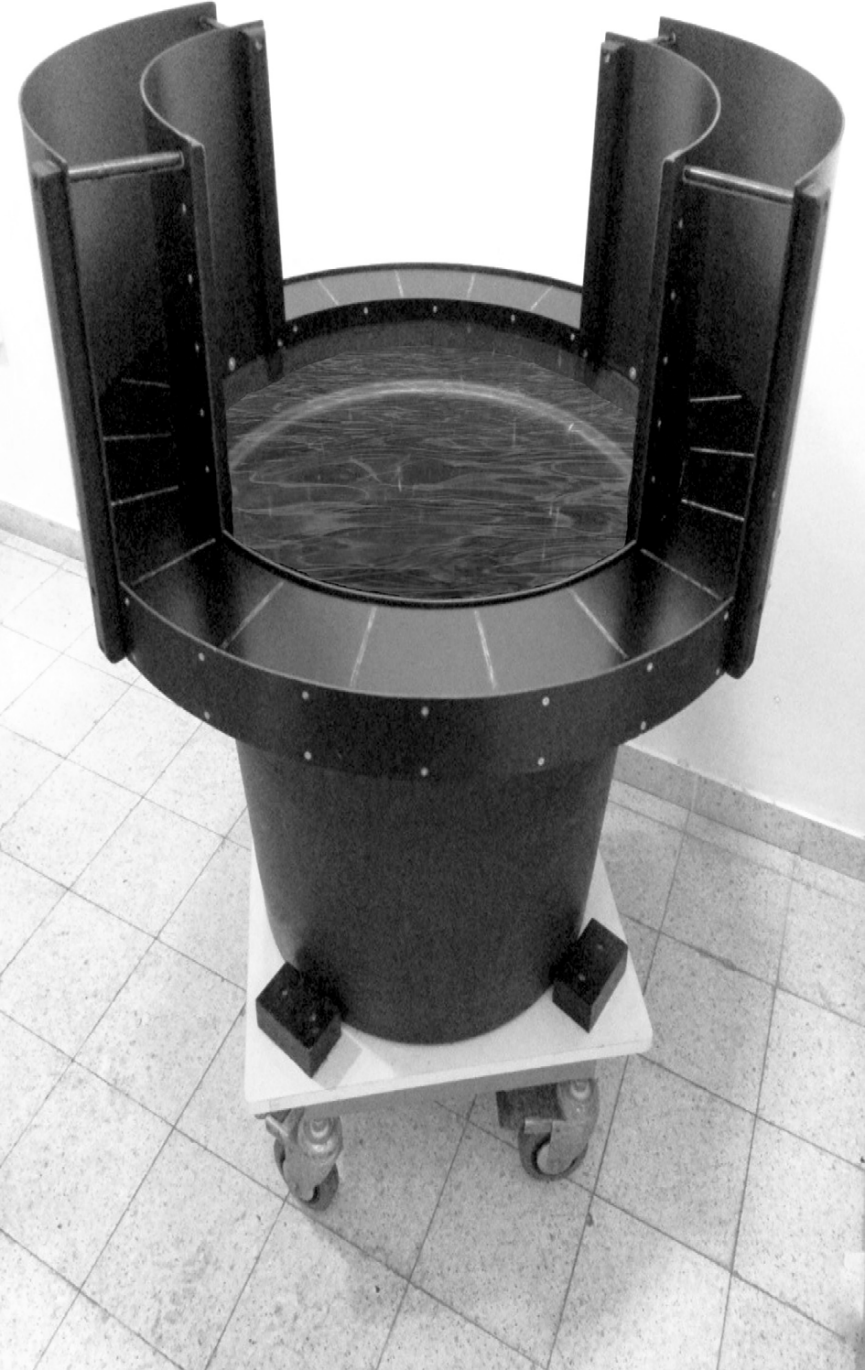
**The water associated zero maze**. An annular platform (90 cm diameter; 10 cm width), made out of black plywood, joined to a plastic tank (70 cm diameter, 55 cm deep) elevating it 55 cm above the ground. The annular platform has two opposite, enclosed quadrants (with walls 35 cm height) and two open quadrants (with borders 5 mm height). The plastic tank that holds this platform is filled up with water (22 ± 2°C, 50 cm deep).

The WAZM is a transformation of the elevated zero maze (EZM) to an integrated wet and dry context. This novel apparatus is constructed from an annular platform (90 cm diameter; 10 cm width), made out of black plywood, joined to a plastic tank (70 cm diameter, 55 cm deep) elevating it 55 cm above the ground. The annular platform has two opposite, enclosed quadrants (with walls 35 cm height) and two open quadrants (with borders 5 mm height). The plastic tank that holds this platform is filled up with water (22 ± 2°C, 50 cm deep), arising to 10 cm below the platform level. Thus, the annular platform and the plastic tank comprise one unified arena (Figure 1). For the tests, rats were first habituated to the room for 4 min and then were placed into one of the open quadrants facing a closed part of the apparatus. Rats were allowed to explore the arena for a 5 mins session. During this time rats behavior was tracked, recorded and analyzed by the Etho-Vision system (Noldus Information Technology, Wageningen, Netherlands). Behavioral measures that were analyzed include the time spent in the open quadrants, distance traveled in the open quadrants, distance traveled in the closed quadrants and total freezing behavior.

### Underwater trauma stress

The UWT stress was carried out in the water tank that was used for the 4 days of swim (i.e., the WAZM). Rats were placed in the center of the WAZM and then were immediately pushed and held under water for 45 s using a special metal net (20 × 10 × 15 cm).

### Experimental design

**Figure 2:**
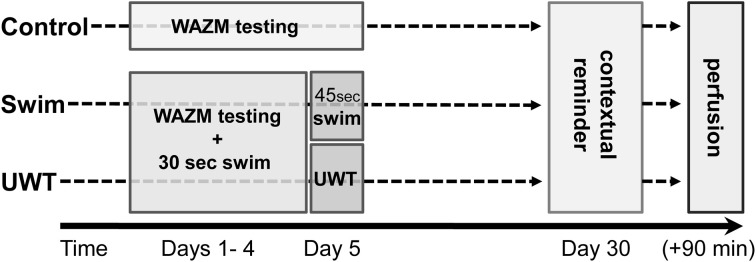
**Experimental design**. UWT and Swim rats were exposed to 4 consecutive days of testing in the WAZM immediately followed by a 30 s swim session. On the 5th day, UWT rats were exposed to the WAZM testing immediately followed by the underwater trauma stress. Swim rats were exposed to the WAZM testing immediately followed by 45 s swim. On the 30th day all rats were exposed to the WAZM testing as a contextual reminder.

Following the acclimation period and random assignment to the different experimental groups (i.e., UWT, Swim, and Control), UWT and Swim rats were exposed to 4 consecutive days of testing in the WAZM immediately followed by a 30 s swim session. On the 5th day, UWT rats were exposed to the WAZM testing immediately followed by the UWT stress. Swim rats were exposed to the WAZM testing immediately followed by 45 s swim. During these 5 consecutive days Control rats were exposed to the WAZM testing only. On the 30th day, 25 days after the last exposure, all rats were exposed to the WAZM testing as a contextual reminder, with no direct exposure to the water (Figure 2).

### c-Fos immunohistochemistry

Ninety minutes after the onset of the contextual reminder, rats were anesthetized with an overdose of sodium pentobarbital (CTS, Israel) and perfused intracardially with ice-cold saline followed by 4% paraformaldehyde in 0.1 M phosphate buffer (pH 7.4; PBS). The brains were removed and stored in the same fixative for 24 h at 4° C, and subsequently immersed in 30% sucrose at 4°C. Brains were than frozen in powdered dry ice and stored at -80° C until sectioning. Coronal sections (30 μm) containing amygdala (approximately -2.0 to -3.15 mm from Bregma) were cut using a cryostat (Leica Microsystems Inc.) at -20° C, and collected in PBS for immunohistochemical processing.

Free-floating sections were washed (3 times for 10 min each) in PBS and incubated for 15 min in Background Sniper (Biocare Medical, USA). Sections were then incubated with the rabbit anti c-Fos primary antibody (1:500 dilution of sc-52, Santa Cruz Biotechnology) in 3% bovine serum albumin (BSA) in PBS with 0. 3% Triton X-100 (PBST) for 24 h on a shaker at 4° C. Sections were again washed in PBS and incubated on a shaker for 1 h with the secondary antibody Alexa Fluor 488-labeled donkey anti-rabbit antibody (1:200 dilution, Cat# A21206, Invitrogen) in PBST at RT. Finally, sections were washed in PBS, mounted on gelatin-coated slides, air-dried, and coverslipped with Gel Mount (Sigma-Aldrich, Switzerland).

Fluorescent images were taken with a Zeiss AxioScope. A1 (Carl Zeiss US) equipped with a digital camera AxioCam MRc (Carl Zeiss US) using a 10X objective. Labeled c-Fos- immunoreactive (IR) nuclei were quantified and averaged from 3 sections in left and right hemispheres for each rat in the 3 groups (Control, Swim, and UWT; *n* = 6 in each group). Sampled areas were defined as whole BLA and CeA regions (about 1 and 0.75 mm^2^ respectively; Figure 4A). The number of c-Fos labeled immunoreactive nuclei was manually counted in a blind manner using ZEN lite 2012 software.

### Statistical analysis

Data are presented as the mean ± standard errors of the mean (s.e.m.). One Way ANOVA with Scheffe *post-hoc* and Paired Samples *t*-test were conducted using SPSS 20 software.

## Results

### Behavior

Twenty-five days after the exposure of UWT rats to the underwater stress, Swim rats to the 45 s swim and Control rats to the WAZM last test, rats were placed back in the WAZM and were allowed to explore the arena for 5 min. During this time rats' behavior was tracked, recorded and analyzed by the Etho-Vision system (Noldus Information Technology, Wageningen, Netherlands).

**Figure 3:**
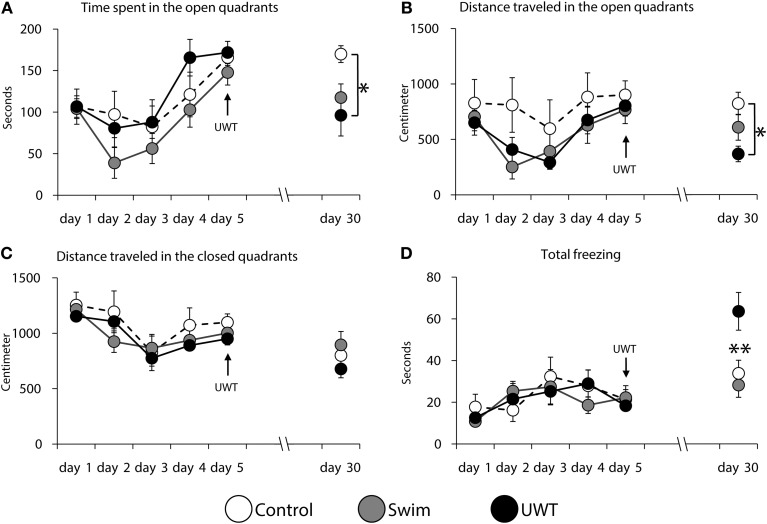
**Rats behavior during the contextual exposure to the WAZM on the 30th day**. Rats that were exposed to the underwater stress spent significantly less time **(A)** and traveled shorter distance **(B)** in the open quadrants of the WAZM. No significant difference was found in the distance traveled in the closed quadrants of the maze between the groups **(C)**. Significant difference was found in total time of freezing in the maze **(D)**. ^*^p < 0.05; ^**^p < 0.01.

Analyses of rats' behavior revealed a significant difference between the groups in time spent in the open quadrants, distance traveled in the open quadrants and total freezing only at day 30. As depicted in Figure 3, time spent in the open quadrants on the 30th day was significantly different between the groups [*F*_(2,23)_ = 4.3, *p* = 0.02]. Scheffe *post-hoc* test further showed that the UWT group (*n* = 8) spent significantly less time in the open quadrants in comparison to the Control group (*n* = 8; *p* < 0.05; Figure 3A). A significant difference between the groups was found for distance traveled in the open quadrants on the 30th day [*F*_(2,23)_ = 4.3, *p* = 0.02]. Scheffe *post-hoc* test further showed that the UWT group traveled significantly less distance in the open quadrants in comparison to the Control group (*p* < 0.05; Figure 3B). A significant difference between the groups was found for total freezing in the WAZM on the 30th day [*F*_(2, 23)_ = 6.7, *p* = 0.009]. Scheffe *post-hoc* test further showed that the UWT group spent significantly more time freezing in comparison to the Control and Swim (*n* = 8) groups (*p* < 0.01; Figure 3D). No significant difference was found in distance traveled in the closed quadrants on the 30th day between the groups (*F*_(2, 23)_ = 1.2, n.s.; Figure 3C). As depicted in Figure 3, no significant differences were found between the groups in any of the measured behaviors during days 1–5.

In order to evaluate which behavior evolved differently in the different groups we conducted Paired Samples *t*-test for the behavioral measures in the WAZM before and after the traumatic experience. As can be seen in Table 1, significant differences in behavior between day 5 and 30 were found only in distance traveled in the closed quadrants among Control rats and distance traveled in the open quadrants among Swim rats. However, when comparing the behavior of UWT rats, before and after the UWT, significant differences were found for all behaviors. These changes indicate a robust reduction in time spent and distance traveled in the open and closed quadrants along with an increase in total freezing 25 days after the exposure to UWT (Table 1).

**Table 1:** **Rats behavior before and after a stressful or traumatic experience**.

	**Control**			**Swim**			**UWT**		
	**Day 5**	**Day 30**	***p***	**Day 5**	**Day 30**	***P***	**Day 5**	**Day 30**	***p***
**TIME (s)**									
Open quadrants	165 ± 10.7	169 ± 10.13	0.424	147 ± 16.8	117 ± 16.4	0.212	171 ± 14.9	95 ± 24.9	0.015
Freezing	22 ± 5.8	33 ± 6.9	0.091	22 ± 5.7	28 ± 5.8	0.376	19 ± 3.6	64 ± 11.1	0.014
**DISTANCE (cm)**									
Open quadrants	899 ± 126	821 ± 113	0.109	765 ± 124	607 ± 116	0.045	802 ± 104	366 ± 78	0.000
Closed quadrants	1097 ± 76	798 ± 90	0.004	1001 ± 94	893 ± 120	0.148	949 ± 56	676 ± 79	0.007

For statistical analysis, Paired Samples t-test was used.

### c-Fos expression

**Figure 4:**
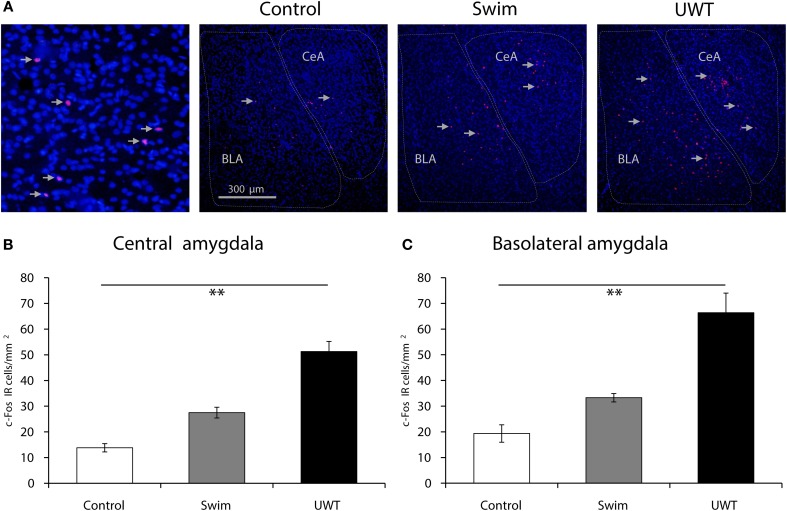
**c-Fos expression 90 min after the WAZM test on the 30th day. (A)** Representative images of c-Fos-IR (red) and DAPI (blue) in the central amygdala (CeA) and basolateral amygdala (BLA). All groups' c-Fos expression differed significantly in the CeA **(B)** and BLA **(C)**. All bars represent the mean ± s.e.m. ^**^p < 0.01.

Quantification of c-Fos IR nuclei revealed increased c-Fos expression in both sub-regions of the amygdala. As depicted in Figure 4, a significant difference between the groups was found for c-Fos expression in the CeA 90 min after the exposure to the WAZM on the 30th [*F*_(2, 17)_ = 46.1, *p* < 0.001]. Scheffe *post-hoc* test further showed that CeA c-Fos expression in UWT rats was significantly higher than the expression in Swim rats, which had significantly higher expression than Control rats (*p* < 0.05; Figure 4B). In regard to the BLA, A significant difference between the groups was found for c-Fos expression 90 min after the exposure to the WAZM on the 30th [*F*_(2, 17)_ = 39.1, *p* < 0.001]. Scheffe *post-hoc* test further showed that BLA c-Fos expression in UWT rats was significantly higher than the expression in Swim rats, which had significantly higher expression than Control rats (*p* < 0.05; Figure 4C).

## Discussion

It was suggested that an optimal animal model of PTSD would mimic the pathophysiological abnormalities and behavioral characteristics of exposure to trauma (Yamamoto et al., 2009). PTSD patients suffer from intrusive memories and re-experiencing of the original traumatic event [DSM-V. American Psychiatric Association (APA), 2013]. These memories are suggested to often be triggered by contextual cues that have become associated with the event in much the same way that the CS are associated with the US in a contextually cued fear memory retrieval (Tronel and Alberini, 2007).

Using a novel behavioral test, the current study demonstrates that exposure to the context that immediately precedes a stressful (e.g., swim) or traumatic (e.g., UWT) events manifest complex behavioral changes. These changes were found in anxious-like behaviors, such as distance travelled and time spent in the “danger” parts of the maze (open quadrants) as well as in freezing behavior. However, no difference between the groups was found in rat's behavior in the “safe” parts of the maze (closed quadrants). Moreover, much like the temporal progression of the human pathology it was meant to model, these changes were measured a long time (25 days) after the actual traumatic or stressful events.

Employing the WAZM protocol leaves it unclear whether the contextual reminder that triggered the traumatic memories was the presence of the water, the context of the WAZM or the combination of the two. Nevertheless, it should be noted that during the test the animals are not directly exposed to the water and of course they do not re-experience the trauma. The ability to identify clear behavioral and amygdala activation effects long time after the actual exposure to the trauma is a strong indicator to the sensitivity of the proposed test.

Another characteristic of the intrusive recollections in PTSD is their resilience to behavioral extinction, which was not directly tested here. However, the very long-term effects of the traumatic but not the stressful exposure, testify for the durability and intensity of the effects of the trauma exposure.

The expression pattern of c-Fos in the CeA and BLA during the retrieval of stressful and traumatic memories found in the present study is consistent with a large body of work suggesting a strong relationship between these regions activation and retrieval of direct associations between contexts and aversive stimuli (LeDoux, 2000; Maren and Quirk, 2004; Fanselow and Poulos, 2005). Furthermore, the results have shown a differentiating pattern of expression in the different groups which significantly differ in accordance to the levels of the original event. Thus, animals that were exposed to an UWT exhibited the highest levels of c-Fos expression in both the CeA and BLA in comparison to animals that were exposed to a swim stress. In accordance, animals that were exposed to a swim stress exhibited higher levels of c-Fos expression in both the CeA and BLA in comparison to control animals.

Within the context of drug development, preclinical animal models of psychiatric disorders have so far failed to serve as effective predictors of candidate drug efficacy (Agid et al., 2007; Markou et al., 2009; Brunner et al., 2012). One contributing factor may be poor dissociation in pre-clinical studies between stressful and traumatic experiences (Koolhaas et al., 2011). Another problem is the lack of detailed enough behavioral profiling that could reflect and relate to the typical individual variability in symptoms in human patients.

The current results suggest that the WAZM can be used to model and measure the long term effects of an exposure to a traumatic event. These measures add to the sensitivity and exactitude of characterization of individual differences and of dissociation between stressful and traumatic experiences. Thus, recruiting the WAZM as part of the battery of behavioral measures in pre-clinical studies of stress-related psychopathologies may contribute to increased predictability of drug testing platforms of these complex disorders.

### Conflict of interest statement

The authors declare that the research was conducted in the absence of any commercial or financial relationships that could be construed as a potential conflict of interest.
